# Draft Genome Sequences of the Lipid-Degrading Bacteria *Moritella* sp. Strains F1 and F3, Isolated from Mesopelagic Seawater from the Sagami Trough, in Japan

**DOI:** 10.1128/MRA.00046-21

**Published:** 2021-08-19

**Authors:** Kei Kagaya, Rika Kogure, Masahiko Okai, Satoshi Kawato, Hidehiro Kondo, Ikuo Hirono, Masami Ishida

**Affiliations:** a Graduate School of Marine Science and Technology, Tokyo University of Marine Science and Technology, Tokyo, Japan; b Department of Ocean Sciences, Tokyo University of Marine Science and Technology, Tokyo, Japan; c Department of Marine Biosciences, Tokyo University of Marine Science and Technology, Tokyo, Japan; University of Southern California

## Abstract

*Moritella* sp. strains F1 and F3 are lipid-degrading bacteria that were isolated from intermediate water from the Sagami Trough, in Japan. We present the draft genome sequences of these two strains, which have 4,983,334 bp and 4,967,310 bp, respectively.

## ANNOUNCEMENT

Lipases (EC 3.1.1.3) are ubiquitous enzymes that catalyze the hydrolysis of ester bonds of triacylglycerol (1–3). Microbial lipases are thermally stable and cost-effective and have broad substrate specificities (4, 5). Recently, lipases derived from microorganisms that inhabit the deep sea (deeper than 200 m) have received a great deal of attention because their ability to withstand low temperatures and high pressure is industrially useful (6). There have been a few reports of microbial lipases derived from seawater deeper than 1,000 m (7–9) but, to the best of our knowledge, there have been no reports that focus on lipases from mesopelagic seawater (depths of 200 to 1,000 m).

To search for a novel mesopelagic microbial lipase, we collected seawater with a temperature of 4.4°C from a depth of 500 m in the Sagami Trough, in Japan (34°40.377′N, 139°48.031′E). The seawater samples were spread on BPG (0.5% fish extract, 0.5% peptone, 0.1% glucose, 0.25% MgSO_4_·7H_2_O, 0.1% KCl, and 1.5% agar)-3% NaCl plates, and the plates were incubated at 10°C for 1 week. The bacteria grown on the agar plates were screened for lipid-degrading ability using BPG-1.5% NaCl plates with 0.1% tributyrin at 10°C, and 83 strains that formed a large halo in 5 days were selected. From these 83 strains, we chose 6 that grew at 4°C and did not grow at 20°C on BPG-1.5% NaCl plates. Finally, the growth rates of the 6 strains at 12.5°C and 15°C were measured by the increase in absorbance at 660 nm in BPG-3.0% NaCl medium using a biophoto recorder (TVS062CA; Advantec, Japan), and we obtained strains F1 and F3, which showed higher growth rates at 12.5°C. The growth rates of F1 were 0.226 h^−1^ and 0.199 h^−1^ at 12.5°C and 15°C, respectively, while those of F3 were 0.234 h^−1^ and 0.213 h^−1^ at 12.5°C and 15°C, respectively. The methods used to determine the 16S rRNA sequences of F1 and F3 are available at https://doi.org/10.6084/m9.figshare.14650365.

Strains F1 and F3 were cultured at 11°C for 24 h on agar plates enriched with Difco marine broth 2216 medium (Becton, Dickinson, USA). Genomic DNA was extracted from each isolated strain using an Isoplant II DNA extraction kit (Nippon Gene, Japan). Paired-end libraries were prepared using a Nextera XT DNA library preparation kit (Illumina, USA) and were sequenced using a MiSeq reagent kit v.2 (300 cycles; Illumina), generating 150-bp paired-end reads. The Illumina reads were quality filtered using Fastp v.0.12.2 and were *de novo* assembled using SPAdes v.3.11.1. The draft genomes were annotated by the DDBJ Fast Annotation and Submission Tool v.1.1.4 (10). Default parameters were used for all software. The draft genome of strain F1 comprised 342 contigs, ranging from 201 bp to 454,119 bp, and that of strain F3 comprised 200 contigs, ranging from 202 bp to 738,218 bp (Table 1). The 16S rRNA gene sequences of strains F1 and F3 were completely identical, and BLASTn searches showed that the 16S rRNA gene sequences of the two strains were 99.67%, 99.14%, and 98.28% identical to those of Moritella marina ATCC 15381 (GenBank accession number NR_040842.1), Moritella japonica DSK1 (GenBank accession number NR_025847.1), and Moritella profunda 2674 (GenBank accession number NR_025381.1), respectively, deposited in DDBJ/EMBL/GenBank.

**TABLE 1:** Genome characteristics of *Moritella* sp. strains F1 and F3

Strain	No. of contigs	Contig *N*_50_ (bp)	GC content (mol%)	No. of reads	Total length (bp)	Genome coverage (×)	No. of coding sequences	No. of tRNAs	No. of rRNAs	GenBank accession no.	SRA accession no.
F1	342	129,962	40.5	796,665	4,983,334	40	4,446	63	3	BLRK00000000	DRR254724
F3	200	281,493	40.5	1,681,062	4,967,310	84	4,466	59	1	BLRL00000000	DRR254725

### Data availability.

The accession numbers of the draft genome sequences and the raw reads for *Moritella* sp. strains F1 and F3 are listed in Table 1.
